# *Onchocerca volvulus*-specific antibody and cellular responses in onchocerciasis patients treated annually with ivermectin for 30 years and exposed to parasite transmission in central Togo

**DOI:** 10.1371/journal.pntd.0010340

**Published:** 2022-05-03

**Authors:** Saskia I. Johanns, Richard G. Gantin, Bawoubadi Wangala, Kossi Komlan, Wemboo A. Halatoko, Meba Banla, Potchoziou Karabou, Adrian JF Luty, Hartwig Schulz-Key, Carsten Köhler, Peter T. Soboslay

**Affiliations:** 1 University Clinics Tübingen, Institute for Tropical Medicine, Eberhard-Karls University, Tübingen, Germany; 2 Onchocerciasis Reference Laboratory, Institut National d’Hygiene, Centre Hospitalier Regional, Sokode, Togo; 3 Institut National d’Hygiene, Lomé, Togo; 4 Centre Hospitalier Universitaire, Université de Lomé, Lomé, Togo; 5 National Onchocerciasis Control Programme, Kara, Togo; 6 Université de Paris, Institut de Recherche pour le Développement, Paris, France; University of Zurich, SWITZERLAND

## Abstract

**Background:**

Annual mass drug administrations (MDA) of ivermectin will strongly reduce *Onchocerca volvulus* microfilariae (mf) in the skin and in the onchocerciasis patients’ eyes. Ivermectin treatment will also affect the expression of immunity in patients, such that activated immune defenses may help control and contribute to clearance of mf of *O*. *volvulus*. Longitudinal surveys are a prerequisite to determining the impact of ivermectin on the status of anti-parasite immunity, notably in risk zones where parasite transmission and active *O*. *volvulus* infections persist.

**Methodology/Principal findings:**

Onchocerciasis patients were treated annually with ivermectin and their *Onchocerca volvulus* antigen (OvAg) specific IgG and cellular responses were investigated before and at 30 years post initial ivermectin treatment (30yPT).

Repeated annual ivermectin treatments eliminated persisting *O*. *volvulus* microfilariae (mf) from the skin of patients and abrogated patent infections. The OvAg-specific IgG1 and IgG4 responses were diminished at 30yPT to the levels observed in endemic controls. Prior to starting ivermectin treatment, OvAg-induced cellular productions of IL-10, IFN-γ, CCL13, CCL17 and CCL18 were low in patients, and at 30yPT, cellular cytokine and chemokine responses increased to the levels observed in endemic controls. In contrast, mitogen(PHA)- induced IL-10, IFN-γ, CCL17 and CCL18 cellular production was diminished. This divergent response profile thus revealed increased parasite antigen-specific but reduced polyclonal cellular responsiveness in patients. The transmission of *O*. *volvulus* continued at the patients’ location in the Mô river basin in central Togo 2018 and 2019 when 0.58% and 0.45%, respectively, of *Simulium damnosum s*.*l*. vector blackflies carried *O*. *volvulus* infections.

**Conclusions/Significance:**

Repeated annual ivermectin treatment of onchocerciasis patients durably inhibited their patent *O*. *volvulus* infections despite ongoing low-level parasite transmission in the study area. Repeated MDA with ivermectin affects the expression of immunity in patients. *O*. *volvulus* parasite-specific antibody levels diminished to levels seen in infection-free endemic controls. With low antibody levels, antibody-dependent cellular cytotoxic responses against tissue-dwelling *O*. *volvulus* larvae will weaken. *O*. *volvulus* antigen inducible cytokine and chemokine production increased in treated mf-negative patients, while their innate responsiveness to mitogen declined. Such lower innate responsiveness in elderly patients could contribute to reduced adaptive immune responses to parasite infections and vaccines. On the other hand, increased specific cellular chemokine responses in mf-negative onchocerciasis patients could reflect effector cell activation against tissue invasive larval stages of *O*. *volvulus*. The annual *Simulium damnosum* s.l. biting rate observed in the Mô river basin was similar to levels prior to initiation of MDA with ivermectin, and the positive rtPCR results reported here confirm ongoing *O*. *volvulus* transmission.

## Author summary

Onchocerciasis is a neglected tropical disease, and a major cause of debilitating skin disease and ocular damage that can lead to irreversible blindness. Annual mass drug administrations (MDA) of ivermectin strongly reduces the load of *Onchocerca volvulus* microfilaria (mf) in the skin and in the patients’ eyes. Evolution of onchocerciasis as a disease is prevented by MDA, but recent studies have shown that *O*. *volvulus* transmission has not been completely interrupted.

Repeated MDA with ivermectin affects immune responses, such that activated immune defenses may enhance clearance of mf of *O*. *volvulus*. Longitudinal surveys are required to determine the impact of ivermectin on the status of immunity, notably in risk zones where parasite transmission and active *O*. *volvulus* infections persist. We examined the changes of *O*. *volvulus* parasite-specific antibody and cellular immune responsiveness in patients treated annually with ivermectin for 30 years. Treatment prevented patent *O*. *volvulus* infections, whilst parasite antigen-specific cytokine and chemokine responses increased but *O*.*volvulus*-specific antibody responses declined. Such decreased antibody levels could weaken antibody-dependent cellular cytotoxic responses to infective and tissue-dwelling *O*. *volvulus* larvae. Strengthened monocyte attracting and activation regulated chemokine responses could enhance effector cell migration and activation against larval stages of O.volvulus, possibly also eliciting resistance to further parasite infections.

## Introduction

In the past 3 decades repeated annual treatments with ivermectin have largely eliminated *Onchocerca volvulus* microfilariae (mf) from the skin and eyes of onchocerciasis patients, thereby preventing the emergence of ocular pathologies [1] and profoundly improving dermal health in affected individuals [2]. By targeting high coverage of ivermectin mass drug administration (MDA) to eligible populations, onchocerciasis intervention programs aim to suppress the transmission of *O*. *volvulus*. Although the symptoms of the disease have largely disappeared, *O*. *volvulus* adult worms survive because single annual doses of ivermectin do not kill adult female *O*. *volvulus* and do not exert embryotoxic or embryostatic effects. Such medication does suppress the release of mf from gravid female *O*. *volvulus* for several months, and this measure must be applied repeatedly until fertile female *O*. *volvulus* either stop reproducing mf or die [3–6]. Following the ivermectin-facilitated reduction of mf load in onchocerciasis patients their cellular anergy reversed, parasite-specific Th1-type responses and serum chemokine levels increased [7–12], and such activated immune responses can lead to clearance of mf of *O*. *volvulus*. After 15 years of repeated ivermectin treatments, parasite antigen-mediated cellular production of Th1-, Th2-type and Treg-type cytokines declined in onchocerciasis patients [13], while their *O*.*volvulus*-specific antibody responses persisted at levels higher than those observed in infection-free endemic controls [7,13,14].

In onchocerciasis and lymphatic filariasis IL-10 is a prominent cytokine mediating immune suppression and cellular hypo-responsiveness to filarial and bystander antigens [15–17]. *O*. *volvulus* antigen-induced IL-10 production will dampen the activity of Th1-type, pro-inflammatory IFN-γ, whilst neutralisation of IL-10 enhances IFN-γ production in patients [18,19]. Stronger IFN-γ responses were observed in *O*. *volvulus* microfilariae negative individuals considered putatively immune to onchocerciasis [20]. In addition, peripheral blood cells from *O*. *volvulus* microfilariae positive patients produced less monocyte and T-cell activating and chemoattracting chemokines MCP-4 (CCL13), TARC (CCL17) and PARC (CCL18), suggesting that persistent *O*. *volvulus* can suppress these chemokines. Following ivermectin treatment in onchocerciasis patients, serum chemokine levels increased, and this may contribute to dermal immune responses along with killing and clearance- of *O*. *volvulus* microfilariae. However, to which extent parasite-specific and innate immune responses may change in onchocerciasis patients with a reduced parasite load and ultimate elimination remains an unexplored issue.

The present study investigated the strength and expression of *O*. *volvulus*-specific immune responses in onchocerciasis patients after 30 years of repeated ivermectin treatments. Cellular production of cytokines, of activation-regulated chemokines, and parasite-specific antibody reactivity were examined, and exposure of the study population to *O*. *volvulus* parasite transmission by infected *Simulium damnosum s*.*l*. vector blackflies was also determined.

### Patients and methodology

#### Ethics statement

At each follow up the aims, procedures and risks of the surveys were explained to all participants in the local language by the medical staff at the Centre Hospitalier Regional (CHR) de Sokodé. Skin snip specimen used in this study were collected from participants who gave oral informed consent. Blood samples used in this study were collected from participants (years 1989 until 2005) who gave oral informed consent, from year 2005 until year 2019 participants provided written informed consent. Formal consent was obtained from the parent or guardian of participants under the age of 18 years. The investigations were authorized by the Ministry of Health in Togo and re-approved every 5–10 years (No.2824/87/MSP-ASCF; No.1999/292/MS/CAB; No.0407/2007/MS/CAB/DGS; No.0060/2013/MS/CAB/DGS) and the Comite de Bioethique pour la Recherche en Santé (No.013/2015/CBRS).

### Survey sites and study participants

Female and male study participants were permanent residents in rural villages in central and northern Togo in West Africa. Surveys were conducted in 1989, 1994, 2004 and 2019 in the villages of Bouzalo (09°06’05"N; 01°02’37"E), Kéméni (09°14’03"N; 01°14’36"E) and Sagbadai (09°04’06"N; 01°04’17"E) in the Région Centrale. All villages are sentinel villages of the National Onchocerciasis Control Program (NOCP) and have received annual mass drug administration (MDA) since 1989 via community-directed distribution of ivermectin (150 μg/kg body weight). All onchocerciasis patients participated in regular surveys conducted by the NOCP. Patients were apparently healthy males and non-pregnant women with a documented history of microfilaria of *O*. *volvulus* in skin biopsies. In 1989 at the first surveys all patients were positive for mf of *O*. *volvulus*. The density of *O*. *volvulus* mf was determined in skin biopsies (mf/mg skin) taken from the right and left hip. Endemic controls from the above sentinel villages who joined the surveys in 1989 and 2019 were never diagnosed as being *O*. *volvulus* mf-positive, and they also received annual ivermectin under the community-directed mass drug administration (MDA).

### Serum collection from ivermectin treated onchocerciasis patients and endemic controls

Blood samples were collected from onchocerciasis patients who participated in regular surveys at baseline (before first ivermectin treatment), at 5 years, 12 years, and 30 years post initial treatment with ivermectin (PT). From the same onchocerciasis patients, sera were collected (paired samples) at baseline from n = 98, at 5yPT from n = 22, at 12yPT from n = 42 and at 30yPT from n = 86 participants. The endemic control (endmCTRL) group participants comprised n = 11 at baseline and n = 22 in 2019 at 30yPT, but were not the same individuals at the two timepoints. They were all permanent residents in the OCP sentinel villages, treated annually with ivermectin at 150 μg/kg, but were never found positive for microfilariae of *O*. *volvulus* in any survey.

The density of *O*. *volvulus* microfilariae (mf) was determined in skin biopsies (mf/mg skin) taken from the right and left hip by means of cornea scleral punch (Holth- or Walser-type). Skin biopsies were immediately weighed and then immersed in 100μl physiological saline in separate wells of flatbottomed microtiter plates and stored at room temperature in a high-humidity atmosphere. Microfilariae were counted by microscopical examination after overnight incubation. Patients were treated annually with ivermectin 150 μg/kg and all patients were followed individually until 30 years post initiation of treatment (PT). At each timepoint of examination all participants gave their informed consent, and for correct and complete understanding explanations were always given in the local language.

### Cellular cytokine and chemokine responses in patients and controls

Blood samples (Table 1) were collected, peripheral mononuclear cells (PBMC) isolated and cells stimulated *in vitro* with *O*. *volvulus* antigen (OvAg) or with mitogen (PHA) for 48 hours. In onchocerciasis patients (OnchoPAT, n = 32) and *O*. *volvulus* mf-negative endemic controls (endmCTR, n = 33) PBMC responses to stimulation were analysed at baseline (beforeIVM) and at 30 years post initial treatment (30yPT). The OnchoPAT group participants examined beforeIVM and 30yPT were the same individuals while the endmCTRL group participants were not the same individuals.

**Table 1:** The leukocyte blood cells, erythrocyte (RBC) and thrombocyte counts, haemoglobin concentration and haematocrit measured in peripheral blood samples from *O*. *volvulus* microfilariae positive onchocerciasis patients’ (OnchoPAT) patients and *O*. *volvulus* microfilariae-negative endemic control (endmCTRL) were surveyed in 1989 prior to initiation of community directed treatment intervention (CDTI) with ivermectin (beforeIVM) and at 30 years post annually repeated ivermectin treatments (30yPT) in year 2019.

	OnchoPAT beforeIVM (n = 32)	endmCTRL beforeIVM (n = 11)	OnchoPAT 30yPT (n = 32)	endmCTRL 30yPT (n = 22)
WBC (min;max)	5,236 (3,350;9,025)	4,536 (3,800;6,800)	4,878 (2,600;8200)	4,702 (2,600;8,200)
Neutrophils %	38 (10;55)	43 (30;52)	42 (23,56)	44 (21;87)
Eosinophils %	16 (3;38)**	1 (0;3)	6 (2;15)	5 (0;10)
Basophils %	0 (0;1)	0 (0;0)	0 (0;5)	0 (0;0)
Lymphocytes %	42 (27;57)	43 (30;52)	48 (34;64)	48 (10;75)
Moncytes %	6 (3;11)**	0 (0;3)	4 (1;6)	3 (0;5)
Haematocrit (%)	43	na	41 (29;58)	40 (11;47)
Haemoglobin (g/dl)	na	na	14 (9,20)	13 (4;16)
Thrombocytes (thousand/μl)	na	na	180.37 (53.0;318.0)	200.73 (63;345)
RBC (million/μl)	na	na	4.58 (1.47;5.94)	4.77 (3.34;6.46)
*O*. *volvulus* mf/mg skin [min;max]	55 (3;248)	0	0	0

** p<0.001 OnchoPAT at befIVM compared to OnchoPAT at 30yPT and endmCTRL at beforeIVM compared to endmCTRL at 30yPT (Wilcoxon rank-sum test)

### Isolation and culture of peripheral blood mononuclear cells (PBMC)

In 1989 and 2019 venous blood samples (10 ml) were collected from the same n = 32 participants for *in vitro* cell culture purposes. A complete blood count including a white blood cell differential was conducted for each participant. Haematological examinations were performed in the central laboratory at CHR. PBMC were isolated from whole blood by Biocoll (Biochrom, Germany) density gradient centrifugation. For this, 9 ml of venous blood was diluted with 9 ml of RPMI (Gibco, UK) supplemented with 100 U/ml penicillin, 100 μg/ml streptomycin and 0.25 μg/ml amphotericin B (Gibco, USA). Diluted blood was layered on Biocoll separation solution and samples were centrifuged for 30 min at 1,600 rpm. The PBMC layer was extracted and transferred into RPMI (as above) and centrifuged for 15 min at 1,600 rpm. This step was repeated twice, and thereafter cell pellets were resuspended in RPMI as described above containing 10% foetal bovine serum (FBS). These freshly isolated PBMCs were cultured *in vitro* at a concentration of 2.5 x 10^6^ cells/ml in 0.5 ml of RPMI (as above) with 10% FBS in 48-well microtiter plates (COSTAR No3548). PBMC were stimulated with the mitogen Phytohemagglutinin (PHA, 5 μg/ml), the *O*. *volvulus* adult worm antigen (OvAg, 35μg/ml), or left unstimulated (negative control). Cell cultures were incubated at 37°C with 5% CO_2_ for 48 hours, and thereafter, cell culture supernatants were harvested and stored at -20°C until further use. Cell culture supernatants from the years 1989 and 1995 were stored at -80°C.

### Cytokine- and chemokine-specific enzyme-linked immunosorbent assay (ELISA)

The cytokine and chemokine concentrations in cell culture supernatants of PBMC were quantified using specific sandwich ELISA (R&D Systems). High binding 96-well microtiter plates (CORNING No3690) were coated with capture antibody diluted in PBS according to the manufacturer’s specifications. The plates were sealed, incubated at room temperature overnight, and subsequently washed three times with washing buffer (PBS with 0.05% Tween20, pH 7.4; Sigma-Aldrich P3563). Blocking reagent diluent (RD; R&D Systems) was added to each well and incubated for 1h at room temperature. The solution was then discarded, and plates washed as above. Next, cell culture supernatants were added to wells and a seven-point standard sample row, prepared as recommended by the manufacturer, was applied. The plates were then sealed and incubated at room temperature for 2h, washed as above, and the detection antibody, diluted in RD according to the manufacturer’s specifications, was added to each well. Plates were incubated at room temperature for 2h, then washed as above, and streptavidin-horseradish peroxidase (Streptavidin-HRP, R&D Systems) diluted in RD was added to each well and incubated for 20 min. After 3 washing steps, the TMB substrate solution (Thermo Fisher Scientific #34021) was added to each well and the colouration was stopped with sulfuric acid. The optical density (OD) was determined at 450 nm in each well using a microplate photometer (BIOTEK EL808). The concentration of cytokines (IL-10, IFN-γ) and chemokines (CCL13, CCL17, CCL18) in cell culture supernatants were calculated in relation to the respective standard curves.

### *O*. *volvulus* (OvAg) antigen-specific antibody ELISA

The IgG1 and IgG4 responses of the study participants were investigated using an antigen extract of adult *O*. *volvulus* (OvAg). Isolation of *O*. *volvulus* and preparation OF adult worm-derived antigen (OvAg) was performed as described previously [21,22]. Nodules containing adult *O*. *volvulus* were surgically removed from patients and adult male and female worms were isolated by collagenase digestion. Freshly isolated worms were extensively washed in sterile phosphate-buffered saline (PBS), transferred into a Ten Broek tissue grinder, and then homogenized extensively on ice. The homogenate was then sonicated twice (30% intensity) for 10 min on ice and centrifuged at 16,000 g for 30 min at 4°C. The supernatants were collected then sterile filtered (0.22 mm) and stored in aliquots at -70C° until use. The protein concentration of the antigen preparation was determined with the BCA protein assay (Pierce, Rockford, USA).

96-well microtiter plates (CORNING No3690) were coated with 50 μl/well of OvAg diluted in PBS (pH 7.4) to a concentration of 5 μg/ml and incubated at 4°C overnight. The OvAg solution was discarded, and plates blocked using 50 μl/well of PBS/0.05%Tween20 (pH 7.4) (Sigma-Aldrich P3563) supplemented with 5% foetal bovine serum (FBS, Therma Fisher Scientific No10270106) for 1.5 h at room temperature. Plates were washed three times using PBS-Tween20 and then 50 μl of serum samples diluted 1:4 in PBS were applied to each well. The samples were incubated for 2 h at 37°C, washed as above and 50 μl of anti-human IgG4 HRP-conjugated monoclonal antibody (Thermo Fisher Scientific A10654) diluted 1:500 in PBS-Tween20 with 5% FBS was added, and plates were incubated for 1.5 h at room temperature. Then plates were washed as described above and TMB substrate solution (Thermo Fisher Scientific #34021) was added to each well, colouration was stopped with sulfuric acid and optical density (OD) was determined at 450 nm in each well using a microplate photometer (BIOTEK EL808).

### Skin biopsy collection

Before ivermectin MDA (delivered by community-directed drug distributors, CDDs), participants gave their informed consent for the collection of skin biopsies to detect *O*. *volvulus* microfilariae (mf). From each participant, a skin biopsy was taken from the left and right iliac crest (for a total of two snips) with a sterile 2-mm Holth corneo-scleral punch biopsy tool. Immediately, skin snips were incubated with physiological saline solution for 30 minutes, and each biopsy was microscopically examined for emerging *O*. *volvulus* mf and their number counted. After this first examination, the two biopsies were transferred separately into a round-bottom well of a 96-well plate containing saline solution, and after 24-hour incubation biopsies were re-examined as before. The use of two incubation steps for skin biopsies is the standard procedure applied by the National Programme for Onchocerciasis Control (PNLO/APOC), and this approach makes it possible to detect *O*. *volvulus* mf which may emerge slowly from skin.

### Blood, stool, and urine sample examination for parasite (co-)infections

For the determination of intestinal helminth and protozoan parasites, fresh stool samples (0.5 g) were mixed with saline and dispersed on 2 microscope slides covered with a 24×48-mm slide; samples were examined by laboratory technicians at the Centre Hospitalier Regional (CHR) de Sokode. All stool samples were examined using the Kato-Katz technique for quantification of helminth eggs per gram stool (helm-TEST; Labmaster). To detect *Schistosoma haematobium* infestation, 10 ml of urine from each participant was filtered (polycarbonate membrane; pore size, 12 μm; Whatman); the filters were then examined under a microscope, and *S*. *haematobium* eggs were quantified. For detection of *Plasmodium spp*. parasites, a thick blood film and a rapid detection test kit was applied (Malaria P.f HRP-II Antigen Rapid Test, Standard Diagnostics Inc., Korea).

### Simulium damnosum s.l. collection

From year 1976 until 2014 the collection of *S*. *damnosum s*.*l*. was conducted at specific catch points at river Mô by trained fly catchers in proximity to the sentinel villages Bouzalo, Sagbadai and Kemeni/Aleheride during the rainy season on five consecutive days in August and September. Collections took place from 7am to 6pm alternating the fly catchers every two hours. Approaching blackflies were caught upon landing and before biting.

In addition, from April 2018 until February 2020, throughout the years weekly collections of *S*. *damnosum s*.*l*. were continued at the river Mô site, in proximity to the villages of Bouzalo and Sagbadai (Region Centrale). The blackflies caught daily were first frozen, then suspended in 70% alcohol, and 25 individual *S*. *damnosum s*.*l*. were pooled into a single tube in ethanol and stored below -20°C until DNA extraction and real-time-PCR (rtPCR). The sampling procedure was the same as above, and this long-term collection was used to determine the annual biting rate (ABR), which was calculated by multiplying the average number of blackflies caught daily by the number of days per week in the month to add up to 12 months.

### Dissection of Simulium damnosum s.l.

The captured *S*.*damnosum s*..*l*. were inactivated at 4°C, dissected using a stereo microscope and classified as parous or nulliparous. The parous flies were divided into head, thorax, and abdomen. Each part was dissected by teasing it apart in a normal saline solution, using dissecting needles and a stereo dissecting microscope, and examined for *O*. *volvulus* larvae. L3 larvae were defined as infective stages present in the head. Dissections were carried out by experienced technicians.

### DNA-Extraction from captured *Simulium damnosum*

For DNA extraction the *S*. *damnosum* samples in ethanol were evaporated overnight, ground in 80μl PBS and the DNA was extracted using the QIAmp DNA Mini Kit (QIAGEN) following the protocol for DNA extraction from tissues. The DNA concentration of each sample was measured by absorbance at 260 nm using the NanoDrop 1000.

### Real-time polymerase chain reaction for Ov33 and Ov150

In blackfly samples *O*. *volvulus* DNA was detected by real-time polymerase chain reaction (PCR) using the MIC qPCR system (BIOZYM, Germany). The *O*. *volvulus* specific gene sequences of Ov33 and Ov150 were selected for qPCR and respective primers and probes were applied as previously described [23,24]. All samples were measured in duplicates. The threshold and Ct values were determined using the MIC qPCR software. The prevalence of Ov33 and/or Ov150 in blackflies pools was calculated as described by Katholi et al. [24].

The following primers and probes were used for the qPCR reaction: Sequence 5’-3’ OV33fwd: GCA AGC TCC AGT TGA AGC AC; OV33rev:CGG CAT TTT CAC GTC CAA GT; OV33probe: FAM-AGA ACC ACC ACA TTT CTG CGT CGC A-TAM; OV150fw: TGT GGA AAT TCA CCT AAA TAT G; OV150rev: AAT AAC TGA CCT ATG ACC; OV150probe: FAM-TAG GAC CCA ATT CGA ATG TAT GTA CCC-TAM

The Ov33 and Ov150 PCR reaction mix was at a total volume 25ul per tube. For Ov33 PCR: Primer Fwd (1.2ul; 10uM); Primer Rev (1.2ul; 10uM); Probe (0.7ul; 10uM); For Ov150 PCR: Primer Fwd (1.2ul; 20uM); Primer Rev (1.2ul; 20uM); Probe (0.7ul; 10uM); for both qPCR: TaqMan 2x Universal MasterMix: (12.4ul); DNA: 5ul; H2O: 4.5ul

The Cycle conditions were for Ov33: Activation (50°C; 2min)(1x); Hold (95°C;10min)(1x); Cycles 60x Denaturation (95°C;10s); Annealing (54°C;30s); and for Ov150: Activation (50°C;2min)(1x); Hold (95°C;10min)(1x); Cycles 60x Denaturation (95°C;15s); Annealing (49°C;30s); Elongation (60°C;2min).

### Analysis of data

The statistical package JMP 14.2.0 was used for statistical analyses. Data obtained with the cytokine and chemokine ELISA were processed using Microsoft Excel 2019. Mean OD values from control wells incubated with RD were subtracted from all sample values measured on the plate. The OD values of the standard samples were used to compile a reference curve and its function was applied to calculate for each cytokine and chemokine the concentrations in pg/ml and multiplied with the dilution factor of the tested samples. Negative values were set to zero. To assess parametric and non-parametric distribution of data the Shapiro-Wilk-W-Test was applied. Data were descriptively explored by ANOVA whereby the level of significance with multiple testing was adjusted according to Bonferroni Holm (PAT and CTRL group, IgG responses, cytokines, chemokine, stimulations, time point of study) which resulted in an alpha level of 0.003. The analysis of the cytokine and chemokine production was performed on logarithmically transformed data. Correlations between cytokines and chemokines as well as age were explored with Spearman correlation coefficient. With multiple comparisons and to avoid type I errors, the OD values and cytokine and chemokine concentrations in the patient and control groups were analysed by the Wilcoxon signed-rank test with level α = 0.003 adjusted.

## Results

### Onchocerciasis patients and endemic controls

At initial registration in 1989 (baseline) the *O*. *volvulus* microfilariae positive onchocerciasis patients’ (OnchoPAT) mean age was 34.7 years (min 16y, max 50y) and 62.4 (45y, 80y) in 2019 (n = 32). The OnchoPAT group participants examined in 1989 and 2019 were the same individuals. The mean age of the *O*. *volvulus* microfilariae-negative endemic control (endmCTRL) group participants in 1989 (n = 11) was 32.8 (20y, 48y) and 43.8 years (27y, 57y) in 2019 (n = 22). The endmCTRL group participants examined in 1989 and 2019 were not the same individuals. In the endmCTRL and OnchoPAT groups 63.6% and 72.4% were male participants in 1989, and 84.4% and 63.6% in 2019, respectively. At baseline (in 1989), eosinophil granulocyte and monocyte percentages were higher in OnchoPAT when compared to endmCTRL (p<0.001 for both cell types); eosinophil counts in OnchoPAT decreased to levels similar to those seen in endmCTRL (p>0.05) at 30yPT. In 1989 the density of *O*. *volvulus* microfilaria (Mf) was 55 mf/mg skin in OnchoPAT (Table 1), while skin biopsies taken at 5yPT, 12yPT and 30yPT contained 0 mf/mg. *Plasmodium falciparum* was the most frequent parasitic infection diagnosed in 28% of OnchoPAT and in 55% of endmCTRL (Table 2). *Entamoeba histolytica* was diagnosed in 17% and 27% and *Necator americanus* in 17% and 23% of OnchoPAT and endmCTRL, respectively. No parasites were detected in urine samples. With respect to parasite (co-)infection, 21 participants (40%) were free of other parasite infections, 24 participants (45%) were singly infected, 6 participants (11%) were infected with 2 parasites, and 3 (6%) with 3 parasites (Table 2).

**Table 2:** Parasite co-infections in onchocerciasis patients (n = 32) and endemic controls (n = 22) surveyed at 30yPT in 2019. The infection-free, singly infected, and poly-parasitized onchocerciasis patients and endemic controls are listed. ^&^Two onchocerciasis patients did not provide stool and urine samples and were tested for *P*. *falciparum* only.

	Onchocerciasis Patients 30yPT (n = 32^&^)	Endemic Controls (n = 22)
*Plasmodium falciparum (*n positive) (%)	9 (28)	12 (55)
*Entamoeba histolytica*	5 (17)	6 (27)
*Entamoeba coli*	4 (13)	4 (18)
Unknown amoeba species	3 (10)	3 (14)
*Necator americanus*	5 (17)	5 (23)
*Giardia lamblia*	1 (3)	2 (9)
*Trichomonas intestinalis*	1 (3)	1 (5)
Co-Infection-free	12 (38)	9 (41)
Single infection	15 (30)	9 (41)
Double infection	2 (7)	4 (18)
Triple infection	3 (10)	0 (0)

### IgG1 and IgG4 antibody response to *O*. *volvulus* adult worm antigen extract (OvAg) in onchocerciasis patients and endemic controls

**Fig 1:**
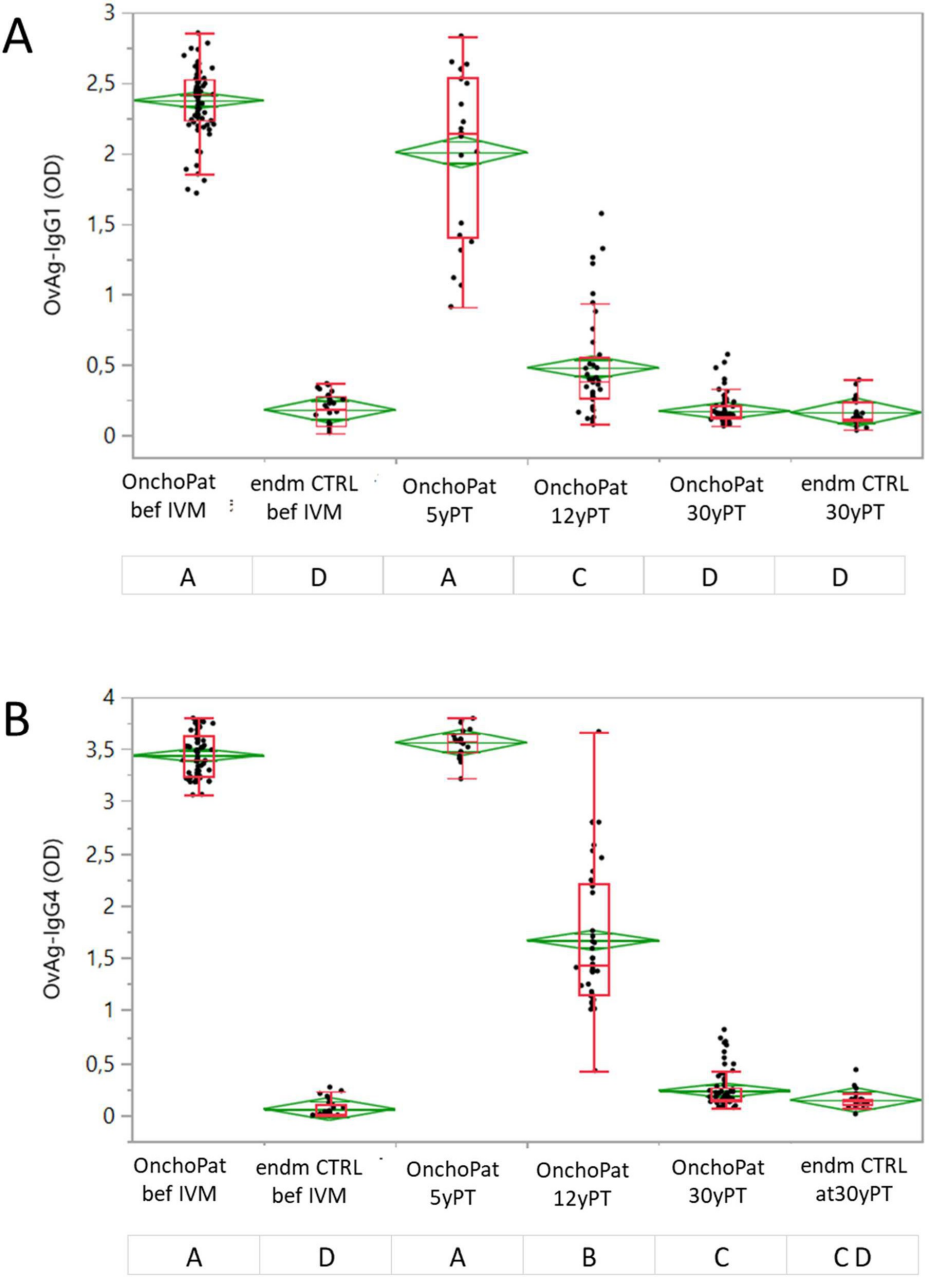
*Onchocerca volvulus* antigen specific IgG1 (**A**) and IgG4 (**B**) responses (OD) in onchocerciasis patients (OnchoPAT) and endemic controls (endmCTRL). The ivermectin treatment was applied annually since year 1989 and the OvAg specific IgG1 and IgG4 responses were investigated in onchocerciasis patients (OnchoPAT) at before ivermectin (beforeIVM, n = 83, 23femaler/60male), 5 years post initial treatment (5yPT, n = 22; 7f/15m), 12yPT (n = 40, 16f/24m) and 30yPT (n = 73; 20f/53m). OvAg-specific IgG1 and IgG4 responses in endemic controls (endmCTRL) were investigate in 1989 (beforeIVM, n = 30, 10f/20m) and at 30 years post initial ivermectin treatment (30yPT; n = 26, 10f/16m). The IgG1 and IgG4 OD values (diamonds) are shown as means with the upper and lower 95% confidence intervals. The data presented in box plots show the median OD values with the 25% und 75% quartiles and the 1.5x of the interquartile range with outliers as individual points. The OD values in the patient and control groups were analysed by the Wilcoxon signed-rank test with the level α = 0.003 adjusted, and groups (time points) not connected with the same letter differ significantly with p<0.0001.

*O*. *volvulus* microfilariae (mf) positive onchocerciasis patients (OnchoPAT) were first examined in 1989, thereafter treated annually with 150μg/kg ivermectin. The endmemic CTRLs were never diagnosed *O*. *volvulus* mf-positive, and they received annually ivermectin under the community-directed mass drug administration (MDA). Parasite antigen (OvAg) specific IgG1 (Fig 1**A**) and IgG4 (Fig 1**B**) responses were studied in OnchoPAT prior to initiation of community directed treatment intervention with ivermectin (beforeIVM) from n = 83, at 5 years post initial ivermectin treatment (5yPT) from n = 22, at 12yPT from n = 40 and at 30yPT from n = 73 participants. At before ivermectin medication, the IgG1 and IgG4 reactivity in OnchoPAT was highly above the endmCTRL group (Fig 1). At 5yPT, the IgG1 responses diminished (p = 0.012) while IgG4 persisted at high levels. At 12 yPT, the OvAg-specific reactivity of both IgG1 and IgG4 diminished in OnchoPAT (p < 0.0001) when compared to before and to 5yPT post initial ivermectin treatment, but responses remained above (p < 0.0001) those observed in OnchoPAT at 30yPT. The IgG1 and IgG4 reactivity at 30yPT was at similar levels in OnchoPAT as observed in endmCTRLs (Fig 1**A** and 1**B**).

### Cellular interleukin-10 and interferon-γ production in onchocerciasis patients and controls

**Fig 2:**
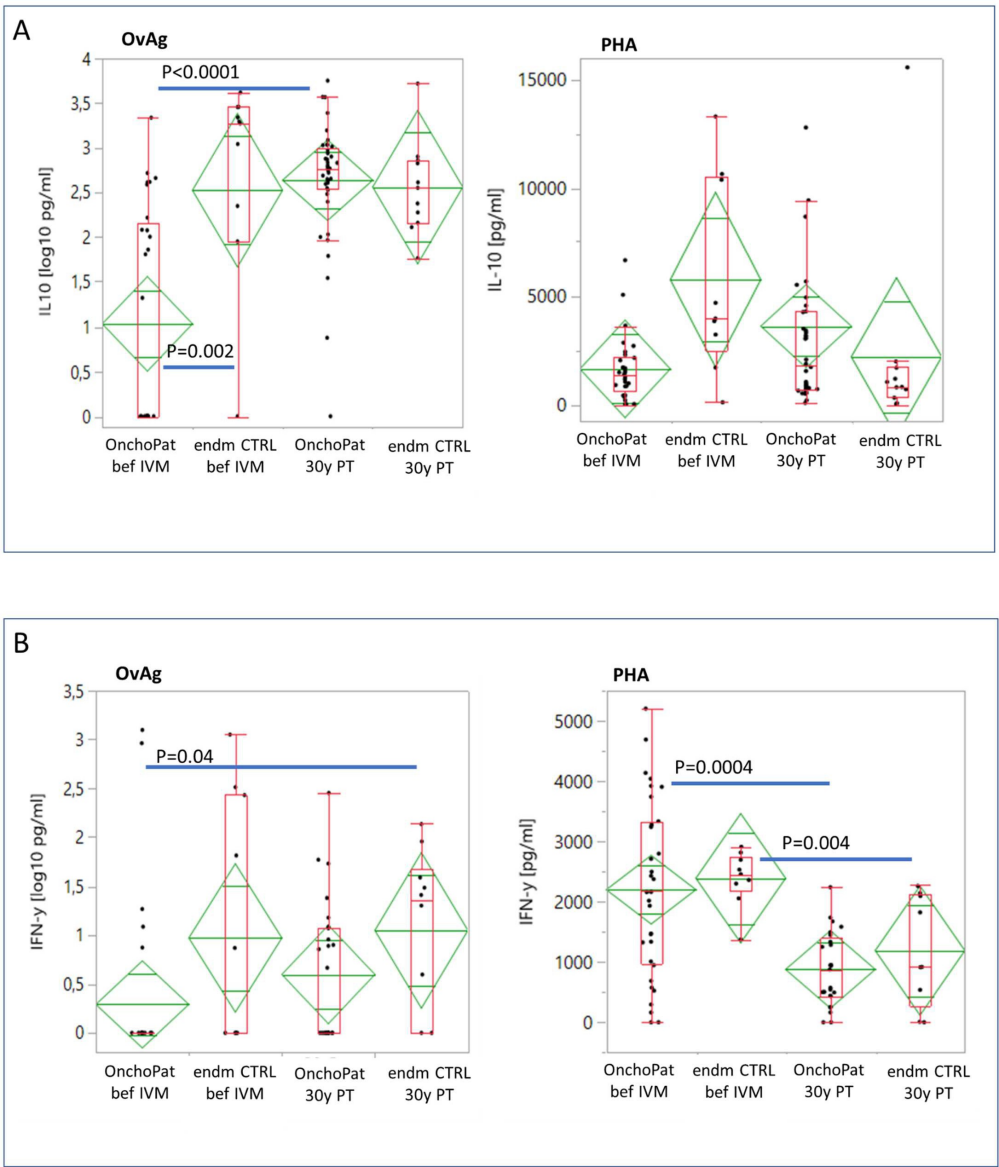
The PBMC production of cytokines IL-10 (**A**) and IFN-γ (**B**) released into cell culture supernatant by peripheral mononuclear blood cells (PBMC) cultured *in vitro* after stimulation for 48 hours with *Onchocerca volvulus* adult worm antigen (OvAg, 35μg/ml) or with the mitogen phytohemagglutinin (PHA, 5μg/ml). The same OnchoPAT were studied at baseline and at 30 years post ivermectin (n = 32, paired) and PBMC were freshly isolated and cultured. PBMC from endm CTRL at baseline (n = 11) and at 30 years post ivermectin (n = 22, unpaired) were similarly processed. Cytokines were quantified with specific ELISA. The data presented in diamonds are the mean cytokine and chemokine productions in pg/ml with the upper and lower 95% confidence intervals. The data presented in box plots show the median production in pg/ml with the 25% und 75% quartiles and the 1.5x of the interquartile range with outliers as individual points. The cytokine and chemokines concentrations are indicated as total amounts without the subtraction of the unstimulated PBMC cultures (baseline). The cytokine concentrations in the patient and control groups were analysed by the Wilcoxon signed-rank test with the α = 0.003 level adjusted.

Peripheral blood mononuclear cells (PBMC) were cultured and stimulated for 48 h with mitogen PHA or *O*. *volvulus* antigen (OvAg). The mitogen PHA strongly increased the IL-10 production in PBMC when compared to OvAg. The spontaneous IL-10 production in unstimulated PBMC cultures was similar at baseline (beforeIVM) and at 30 years post initial treatment (30yPT) in OnchoPAT and EndmCTRL. At baseline the OvAg-induced IL-10 production was significantly lower in OnchoPAT (175pg/ml) than in endmCTRL (1,587pg/ml) (p = 0.002) (Fig 2**A**). At 30yPT, the induced IL-10 production increased in OnchoPAT (p < 0.0001) to similar levels as observed in endemic controls (913pg/ml and 588pg/ml, respectively). The activation of PBMC in OnchoPAT with mitogen PHA resulted 1,626pg/ml of IL-10 which was less than in endmCTRL (5,758pg/ml), and at 30yPT, higher amounts of IL-10 were secreted by cells from OnchoPAT (3,815pg/ml) than endmCTRL (2,261pg/ml).

OvAg antigen did not activate the production of IFN-γ in PBMC from OnchoPAT at baseline (min-max: 0–20 pg/ml), and OvAg inducible IFN-γ production was at similar levels with 19 pg/ml (min-max: 0-38pg/ml) at 30yPT. Higher amounts of IFN-γ were released in cell culture supernatants from endmCTRL at baseline with 67 pg/ml (min-max: 33–100 pg/ml) and IFN-γ was lower at 30yPT with 31 pg/ml (min-max: 8–53 pg/ml)(Fig 2**B**).

With mitogen PHA stimulation the IFN-γ cellular release increased above the baseline production significantly. The PHA-induced IFN-γ production by PBMC was similarly high in patients (2,200pg/ml) and endemic controls (2,391pg/ml) at baseline, and IFN-γ levels diminished in OnchoPAT (877pg/ml) (p = 0.0004) and in endmCTRL (1,189pg/ml) (p = 0.004) to similar levels at 30yPT (Fig 2**B**).

### Chemokines

#### CCL13 (MCP-4), CCL17 (TARC) and CCL18 (PARC)

**Fig 3:**
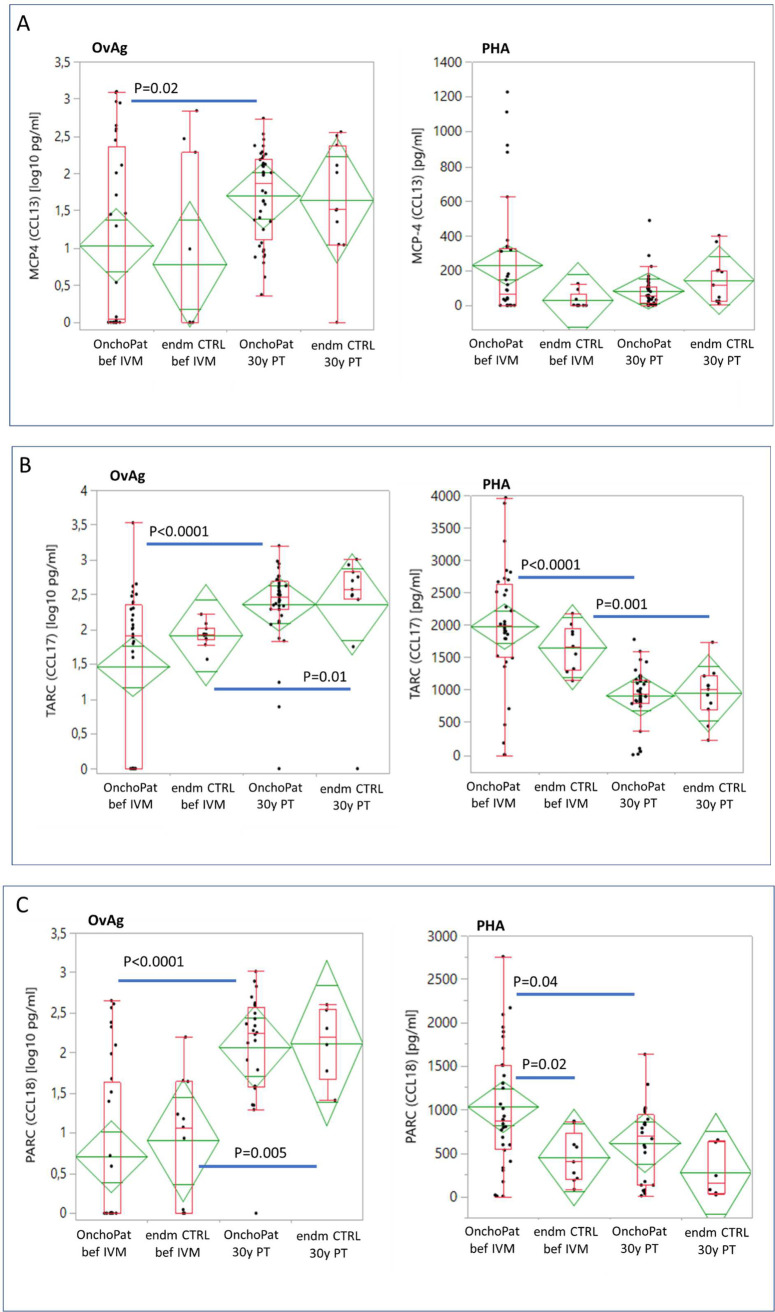
The *in vitro* production of CCL13 (MCP4) (**A**), CCL17 (TARC) (**B**) and CCL18 (PARC) (**C**) by PBMC stimulated for 48 hours with *Onchocerca volvulus* adult worm antigen (OvAg, 35μg/ml) or with the mitogen phytohemagglutinin (PHA, 5μg/ml). The same onchocerciasis patients were studied at baseline and at 30 years post ivermectin (n = 32, paired) and PBMC were freshly isolated and cultured. PBMC from endemic controls at baseline (n = 11) and at 30 years post ivermectin (n = 22, unpaired) were equally applied. Chemokines were quantified with specific ELISA. The chemokine concentrations in cell culture supernatants are shown in diamonds as means with the upper and lower 95% confidence intervals. The data presented in box plots show the median in pg/ml with the 25% und 75% quartiles and the 1,5x of the interquartile range with outliers as individual points. The cytokine and chemokines concentrations in cell culture supernatants are indicated as total amounts without the subtraction of the unstimulated PBMC cultures (baseline). The chemokine concentrations in the patient and control groups were analysed by the Wilcoxon signed-rank test with the α = 0.003 level adjusted.

The cellular production of CCL13 by PBMC stimulated with OvAg and mitogen PHA is shown in Fig 3**A**. At baseline, the spontaneous (baseline) secretion of CCL13 from not activated PBMC was 146pg/ml in OnchoPAT and 68pg/ml in endCTRL, and at 30yPT, baseline CCL13 levels diminished to 74pg/l and 105pg/ml, respectively. At baseline, the CCL13 secretion in response to OvAg increased slightly both in patients (192pg/ml) and endemic controls (108pg/ml). At 30yPT, the OvAg-induced cellular CCL13 production augmented in OnchoPAT (p = 0.02) to similar levels as in the endmCTRL group. The PHA stimulated CCL13 production was highest at baseline in patients and diminished from 230pg/ml to 92pg/ml at 30yPT (Fig 3**A**) while in endemic controls the CCL13 levels augmented from 24pg/m to 124pg/ml (not significant), respectively.

The spontaneous CCL17 release (baseline) by PBMC from patients was highest at baseline with 406pg/ml and reduced to 113pg/ml at 30yPT (p < 0.001) while in endemic controls the baseline CCL17 production was at similar levels at both time points of study (Fig 3**B**). At baseline, the addition of OvAg to PBMC cultures from patients depressed their CCL17 release below the levels measured in unstimulated cells (baseline). Such suppressive effect of OvAg on the cellular production of CCL17 was not observed at 30yPT. PBMC from OnchoPAT stimulated with OvAg released 223pg/ml and at 30yPT higher levels of CCL17 with 392pg/ml were produced (p < 0.0001) (Fig 3**B**). Similarly, in endemic controls the CCL17 levels in cell culture supernatants augmented from 87pg/ml at before ivermectin to 400pg/ml at 30yPT (p = 0.01).

The mitogen PHA induced in PBMC from OnchoPAT the production of CCL17, and its levels were at mean 1,976pg/ml at baseline and diminished to 945pg/ml at 30yPT (p < 0.0001). Similarly, in endmCTRL the CCL17 production by PBMC lessened from mean 1,658pg/ml to 1,092pg/ml at 30yPT (p = 0.001). At 30yPT, the cellular CCL17 production was at equal levels in patients and endemic controls (Fig 3**B**).

At baseline, CCL18 production by PBMC from patients was at 312pg/ml and diminished to 63pg/ml (p < 0.0001) when OvAg was added, while in endemic controls, OvAg did not suppress CCL18 (Fig 3**C**). At 30yPT, the OvAg-induced production of CCL18 increased significantly in OnchPAT (p < 0.0001) and endmCTRL (p = 0.005) and their PBMC released similar amounts of CCL18 into cell culture supernatants. At baseline, the mitogen PHA stimulated CCL18 production was 1,026pg/ml in OnchoPAT 444pg/ml in endmCTRL (p = 0.02) (Fig 3**C**). At 30yPT, the PHA induced cellular CCL18 release into cell culture supernatants lessened in OnchPAT (p = 0.04) and was lowest in endmCTRL when compared with baseline (Fig 3**C**)

### Correlation of cytokines and chemokines

In onchocerciasis patients at baseline, the OvAg-induced cytokine IL-10 production correlated positively with IFN-γ (R = 0.371, *P* = 0.0022), with CCL17 (R = 0.471, *P* < 0.0001) and with CCL18 (R = 0.322, *P* = 0.008) (Table 3). The activation regulated chemokines CCL17 and CCL18 were positively correlated (R = 0.628, *P* < 0.0001). The IFN-γ production correlated only with the the pulmonary and activation regulated chemokine CCL18 (PARC) (R = 0.783, *P* = 0.0009) in the endCTRL group.

**Table 3:** Pairwise correlations of cytokines and chemokines. The significant correlations between the OvAg-induced cytokine and chemokine productions in onchocerciasis patient (OnchoPAT: Bef-6moPT, 30yPT) and *O*.*volvulus* negative endemic controls (endmCTRL) are listed with the coefficient R and the p-value; not significant correlations were omitted.

		OnchoPAT Bef-6moPT		OnchoPAT 30yPT		endmCTRL	
Variable 1	Variable 2	R	p-value	R	p-value	R	p-value
IL-10	IFN-γ	0.3708	0.0022				
IL-10	CCL13	0.2827	0.0214				
IL-10	CCL17	0.4711	<0.0001	0.3424	0.0328		
IL-10	CCL18	0.3223	0.0083				
IFN-γ	CCL13						
IFN-γ	CCL17						
IFN-γ	CCL18					0.7831	0.0009
CCL13	CCL17	0.3052	0.0102	0.4468	0.0044		
CCL13	CCL18	0.3040	0.0105				
CCL17	CCL18	0.6288	<0.0001				

### *Simulium damnosum s*.*l*. human biting rates, annual transmission of *Onchocerca volvulus* infective larvae (L3) and prevalence of *O*. *volvulus* microfilariae (mf) positive human cases in the study area

From 1976 until 2014, the annual biting rate (ABR) and the annual transmission potential (ATP) of infective larvae (L3) of *O*. *volvulus* by *S*. *damnosum s*.*l*. in the Mô river basin were investigated by means of human landing catches and microscopic detection of L3 in captured black flies (Table 4). The control of *S*.*damnosum s*.*l*. through larvicide application by the Onchocerciasis Control Program gradually diminished the ABR and ATP in the Mô river basin, and, until 2014, larviciding, together with ivermectin MDA which began in 1988, reduced the annual transmission of L3 of *O*. *volvulus* by more then 90%. In parallel, the prevalence of *O*. *volvulus* microfilariae in skin biopsies was repeatedly evaluated in the Mô river basin village populations of Bouzalo, Kemeni and Sagbadai from 1976 onwards. through to 2000, the prevalence of mf-positive human cases decreased markedly from 63.5% to 7.5% in Bouzalo, from 53.4% to 0% in Kemeni and from 75.3% to 1.4% in Sagbadai (Table 5).

**Table 4:** Annual biting rates (ABR) by *Simulium damnosum s*.*l*. and the annaul transmission potential (ATP) of *O*. *volvulus* infective larvae (L3). The ABR and the ATP is shown from 1975 until 2014 in the Mô river basin. The villages Bouzalo and Kemeni are situated less than 2 km from the Mô river. The ABR and ATP were determined annually from 1976 onwards during the anti-*Simulium damnosum s*.*l*. intervention activites of the National Onchocerciasis Control Programme (NOCP). The ATP of infective L3 larvae of *O*. *volvulus* by *S*. *damnosum* was determined after microscopic dissection of the captured black flies. Mass drug administration (MDA) with ivermectin began in 1988 and continued after OCP closure in 2002. Special interventions in the post-OCP period included continued aerial larvicide application for five additional years (2003–2007) and twice-yearly ivermectin MDA.

	Mô / Bouzalo		Mô / Kéméni	
Year	ABR	ATP	ABR	ATP
		O.volvulus L3		O.volvulus L3
1976	39.210	667	22.557	155
1977	41.899	1697	21.619	405
1978	32.294	1173	7.818	68
1979	30.673	938	14.281	193
1980	24.286	1062	8.739	n.a.
1981	10.620	n.a.	2.441	0
1982	17.149	382	3.921	64
1983	18.484	200	2.705	18
1984	27.993	622	9.096	80
1985	12.458	401	7.471	83
1986	19.475	358	13.102	338
1987	7.695	n.a.	11.638	64
1988	1.677	12	4.794	43
1989	2.096	12	4.157	33
1990	6.925	30	2.370	0
1991	7.619	96	4.525	10
1992	7.455	0	2.685	0
1993	5.535	0	3.475	0
1994	5.803	10	4.898	0
1995	7.410	23	4.946	71
1996	4.948	0	3.915	15
1997	8.805	165	4.648	21
1998	2.940	15	5.965	0
1999	980	0	3.245	20
				
2013	1147	4		
2014	1491	6		

**Table 5:** Repeated surveys on *Onchocerca volvulus* infestation in villages Bouzalo, Kemeni and Sagbadai. The prevalence of *O*. *volvulus* microfilariae (mf) in skin biopsies in study populations was determined from before mass drug administration (MDA) of ivermectin and several years with the annually repeated MDA. The mass drug administration (MDA) with ivermectin began in 1988 and continued after Onchocerciasis Control Program (OCP) closure in 2002. In the post-OCP period in the Mô river basin special interventions continued with aerial larvicide application for five additional years (2003–2007) and MDA with ivermectin implemented twice annually. The surveyed years, number of examnied participants and the prevalence of *O*.*volvulus* mf in skin biopsies are shown.

Village	Date	No. Examined	Prevalence Mf (%)
			
Kemeni	1976–11	547	53,4
	1980–03	165	49,3
	1982–11	256	46,1
	1985–01	547	35,4
	1986–04	216	27,5
	1996–06	111	4,5
	2000–05	245	0
	2014	364	0
			
Bouzalo	1985–04	212	63,5
	1989–03	141	68,1
	1990–11	119	52,9
	1993–03	427	7,5
			
Sagbadai	1980–05	110	75,3
	1987–01	65	65,5
	1993–03	27	51,8
	1996–06	108	28,2
	2000–05	133	14,3
	2007–04	124	1,4

### Onchocerca volvulus Ov150 and Ov33 DNA in the vector Simulium damnosum s.l.

**Fig 4:**
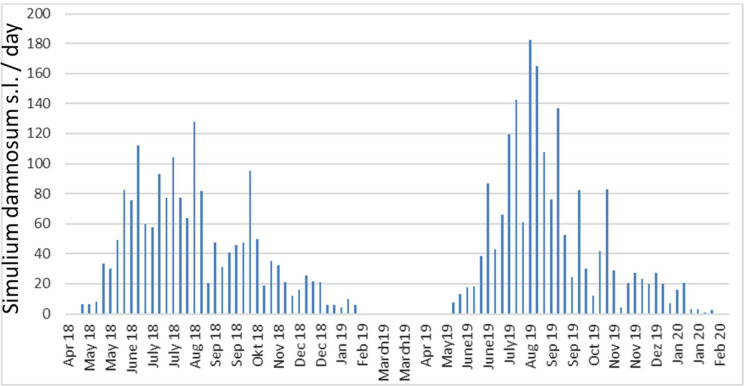
*Simulium damnosum s*.*l*. collection. At specific catch points at river Mô, in proximity to the village Bouzalo, the collection of *S*. *damnosum s*.*l*. was performed weekly by trained fly catchers from April 2018 until February 2020. Bouzalo village is situated less than 2 km from the Mô river. Collections took place from 7am to 6pm alternating the fly catchers every two hours. This long-term collection was to determine the annual biting rate (ABR), which was calculated by multiplying the average number of blackflies caught daily by the number of days per week in the month to add up to 12 months.

From April 2018 until December 2019 *S*. *damnosum s*.*l*. were captured (n = 6,350) (Fig 4) and grouped in 254 pools each with 25 blackflies. In 2018, the annual biting rate (ABR) was n = 12,145 bites/person/year (Fig 4) and the prevalence of Ov150 DNA in Simuliidae was 0.827% (Table 6). In 2019, the ABR was n = 12,688 bites/person/year (Fig 4) and Ov150 DNA was detected in 0.454% (Table 6) of the Simulidae. Biting rates and proportion of infected flies were highest in December of each year. In parallel, the presence of Ov33 DNA was investigated in the captured Simulidae and 1.399% and 1.06% of them were found positive in 2018 and 2019, respectively (Table 6).

**Table 6:** Number of *Simulium damnosum s*.*l*. pools analysed by qPCR in year 2018 and 2019 for the presence of *O*. *volvulus* specific Ov33 and Ov150 DNA. Blackflies were grouped into pools of 25 and the number of Ov33 or OV150 negative and positive pools is shown.

Collection Month	Pool (n) (Neg;Pos)	Prevalence (%)	Pool (n) (Neg;Pos)	Prevalence (%)
	**Ov150**		**Ov33**	
Aug 18	20 (17;3)	0,648	20 (13;7)	1,708
Sep 18	13 (11;2)	0,666	13 (9;4)	1,460
Oct 18	15 (13;2)	0,571	15 (12;3)	0,889
Nov 18	10 (7;3)	1,417	10 (7;3)	1,417
Dec 18	6 (4;2)	1,609	6 (4;2)	1,609
**Total 2018**	**64 (52;12)**	**0,827**	**64 (45;19)**	**1,399**
Jan 19	4 (3;1)	1,144	4 (3;1)	1,144
Feb 19	1 (1;0)	0,000	1 (1;0)	0,000
May 19	1 (1;0)	0,000	1 (1;0)	0,000
June 19	13 (12;1)	0,320	13 (10;3)	1,044
July 19	30 (28;2)	0,276	30 (25;5)	0,727
Aug 19	53 (49;4)	0,313	53 (40;13)	1,119
Sep 19	22 (19;3)	0,585	22 (16;6)	1,266
Oct 19	20 (18;2)	0,421	20 (18;2)	0,421
Nov 19	6 (5;1)	0,727	6 (3;3)	2,735
Dec 19	8 (5;3)	1,863	8 (5;3)	1,863
**Total 2019**	**158 (141;17)**	**0,454**	**158 (121;37)**	**1,061**

The monthly biting rate (MBR) in 2019 peaked in the rainy season months of July, August, and September, while no blackflies were caught in March and April. The Ov33 positive blackflies were most prevalent in November 2019 with 2.73% and least in October 2019 with 0.42%. Most Ov150 positive blackflies were found in December 2019 with 1.86% and 1,609% in December 2018 (Table 6). In February, all *S*. *damnosum* captured tested negative for Ov33 and Ov150. December followed by November and January were the months with the highest prevalence of Ov150 positive blackflies both in 2018 and 2019.

## Discussion

### Ivermectin treatment

Ivermectin will achieve a drastic elimination of *O*. *volvulus* mf from the skin and a long-lasting suppression of microfilaridermia. Ivermectin primarily affects the motility of mf of *O*. *volvulus* and obstructs their release from gravid adult worms for several months [3–6,8]. Adult *O*. *volvulus* worms can live in infected humans within the nodules for over 15 years, necessitating a prolonged treatment course of ivermectin. Owing to the short lifetime of ivermectin and the absence of macrofilaricidal effects ivermectin treated *O*. *volvulus* infected and skin mf-negative individuals will harbour living adult worms. After ivermectin treatment fully developed intra-uterine mf will not be able to actively emerge from the uterus of female *O*. *volvulus* and will degenerate in utero. In adult female worms the intrauterine mf production and mf release is reduced, but reversible 9–12 months after ivermectin treatment. Reproduction of the parasite continues with regular insemination and normal embryonic development, and since this process is not synchronized several gravid female *O*. *volvulus* with different developmental larval stages can be found in the same nodule, and mf will continue to emerge. Thirty years of repeated ivermectin medications permanently eliminated mf of *O*. *volvulus* in the OnchoPAT group, and haematological parameters as well as parasite (co-)infections were similar in the OnchoPAT and endmCTRL groups. The successful application of ivermectin can reduce multiple endo- and ectoparasitic infections, including scabies, pediculosis and soil transmitted helminths. The recently recommended albendazole-ivermectin combination treatment in disease control programs may be superior in terms of cure rates, in reducing infection intensities and preventing parasite transmission.

### IgG1 and IgG4 responses to O. volvulus antigen (OvAg)

The OvAg used in our work was an adult worm extract which contains microfilariae and adult female and male *O*. *volvulus*. Antibody responses until 12 years PT in the OnchoPAT group were mainly directed against microfilariae that continued to emerge from gravid female *O*. *volvulus*. Thereafter, also with the reduced parasite transmission, and annual retreatment with ivermectin the reproductive activity of adult female *O*. *volvulus* reduced and antibody reactivity to OvAg declined.

30 years post initiation of MDA with ivermectin IgG4 and IgG1 responses to *O*. *volvulus* antigens in onchocerciasis patients were at similarly low levels to those measured in endemic (persistently uninfected) controls. Earlier, at 5yearsPT, IgG1 responses had declined slightly while IgG4 reactivity remained high, and this was presumably due to persistent viable adult worms or continuous exposure to infective larvae of *O*. *volvulus* in the study area in central Togo. In chronic filarial diseases, specific IgG4 antibodies make up to 95% of the IgG response [25], requiring repeated antigen stimulation [26], and production of IgG4 is favoured by the cytokines IL-4, IL-13 and IL-10 released by Th2- and Treg-cells. 12yPT all patients were mf-negative and their IgG4 reactivity to OvAg had moderately decreased, although responses remained higher than in endemic controls and above those in post patent onchocerciasis patients [13,14,27]. A single annual dose of ivermectin at 150μg/kg will not kill adult female *O*. *volvulus*, and antigens released from non-reproductive or moribund adult *O*. *volvulus* enclosed in granulomas may have stimulated such antibody production [3–6]. After 30 years of MDA, IgG4 responses in patients had declined to endemic control levels. The IgG4 responses to OvAg increased in onchocerciasis patients over time, possibly due to continuous exposure to filarial antigens [27,28] and repeated trickle infections with *O*. *volvulus* infective larvae.

To assess *O*. *volvulus* transmission, testing of IgG4 responses to Ov16 is recommended by the WHO in children aged <10 years [29], but in higher age groups the IgG4 responses to Ov16 are less sensitive than the IgG4 reactivity to the *O*. *volvulus* adult worm extract [22]. The analysis of skin biopsies for the detection of *O*. *volvulus* DNA by PCR can provide further information on the disease status of survey participants but such an invasive procedure is poorly accepted, notably by children. The IgG4 responses to Ov16 may not suffice as a sole biomarker for the detection of an active *O*. *volvulus* infection and the application of multiple biomarkers is needed to improve sensitivity [30].

### Cellular IL-10 and IFN-γ production

In onchocerciasis and lymphatic filariasis IL-10 is a prominent cytokine mediating immune suppression and cellular hypo-responsiveness to filarial and bystander antigens [31–33]. In patients at 30yPT, the OvAg-specific IL-10 increased and thus may have dampened the Th1-type and pro-inflammatory cytokine IFN-γ. Suppression of IFN-γ release is mediated by an increased production of IL-10 [18], neutralisation of IL-10 will enhance the IFN-γ production in patients [19] and inducible IFN-γ responses were observed in individuals putatively immune to *O*. *volvulus* [20]. In patients at 30yPT, the OvAg inducible IFN-γ production did not attain the levels detected in endemic controls. This may be due to residual adult *O*. *volvulus* which stimulated IL-10 and suppressed IFN-γ production. Such inhibited activation of macrophages, of NK cells, of MHC I and MHC II expression [34] will not activate cellular effector mechanisms, and this can facilitate parasite persistence. In onchocerciasis patients spontaneous cellular IL-10 production has been observed [35], and mf and antigens emerging from adult *O*. *volvulus* and concurrent helminth infections may stimulate IL-10 [36]. The IL-10 responses restored with the ivermectin-supported permanent clearance of *O*. *volvulus* mf, and a similar increase of IL-10 production has been previously observed in onchocerciasis patients treated with ivermectin for 16 years [13]. Despite the age difference between the groups the IL-10 production levels were similar in patients and controls. The positive associations of IL-10 in OnchoPAT at baseline with all studied cytokines and chemokines disclosed its broad regulatory function on T-helper-cell subsets and highlighted IL-10 as a regulator of cellular responsiveness [37]. IL-10 production appears favourable for parasite persistence [35] and T-helper-cell responses regulated by IL-10 and TGF-β may dampen proinflammatory Th1- and Th2-cells, thereby preventing pathogenic cellular hyperreactivity in OnchoPAT [31,32]. In contrast, Th1-type IFN-γ production to OvAg stimulation did not correlate in OnchoPAT with Th2-type chemokines CCL13, CCL17, CCL18 suggesting that their responses were not linked. The Th2-type chemokines CCL13, CCL17 and CCL18 mediate attracting effects on macrophages, monocytes, and eosinophil and basophil granulocytes [38], and their levels are particularly elevated in inflammatory Th2-type responses with various skin diseases [39]. With permanent *O*. *volvulus* mf clearance at 30yPT only regulatory IL-10 and monocyte chemoattractant CCL13(MCP4) remained positively associated with TARC(CCL17), reflecting a persistent Th2-type bias. In the endemic CTRL group IFN-γ responses were associated with the activation regulated chemokine CCL18, revealing a pro-inflammatory Th1-type response capability not evident in the Oncho PAT group.

### Celllar chemokine CCL13, CCL17 and CCL18 production

Before initiation of ivermectin treatment, OvAg-stimulated PBMC produced less monocyte and T-cell chemoattracting MCP-4 (CCL13), TARC (CCL17) and PARC (CCL18) suggesting that persistent *O*. *volvulus* can suppress these chemokines. 30yPT, the OvAg-inducible CCL13, CCL17 and CCL18 release was similarly increased in patients and endemic controls showing that, with permanent elimination of mf of *O*. *volvulus*, chemokine responses were re-activated, and, that an age-related decline of the cellular chemokine production capacity had not developed. As previously observed in onchocerciasis patients, the serum concentrations of MCP-4 (CCL13) increased 3 days post initial ivermectin treatment and this may signify ongoing effector cell activation against microfilariae of *O*. *volvulus* [11].

The chemokine MCP-4 mediates selective recruitment and activation of effector cells to inflamed tissues [40,41], and with permanent elimination of mf at 30yPT the cellular production of MCP-4 was increased to control levels. Similarly, OvAg-stimulated CCL17 (TARC) and CCL18 (PARC) production increased significantly. The activation regulated chemokine CCL17 (TARC) attracts Th2- and Treg-cells and will direct their homing to the skin [42–44]. TARC is a serological indicator of multiple helminth infections [45,46] and higher CCL17 levels reflect immune activation of basophils, eosinophils, and macrophages. High levels of activation regulated CCL18 are present in human plasma and increased CCL18 production has been demonstrated in several diseases, including malignancies and inflammatory joint, lung, and skin diseases [47,48]. 30yPT, OvAg-stimulated cellular CCL17 and CCL18 productions were at similar levels in patients and endemic controls, suggesting that cytokine and chemokine production capacities were re-established in patients. In contrast, mitogen-induced polyclonal cellular responses diminished with expiring *O*. *volvulus* infections. This divergent response profile suggests strengthened parasite-specific cellular reactivity while innate immune responses were dampened. The onchocerciasis patient group (mean age 62.4 years) exhibited a reduced innate mitogen-inducible cellular production of the Th1-type cytokine IFN-γ and of the activation regulated chemokines TARC and PARC, while IL-10 responses increased. Such altered innate immune responsiveness in the elderly patients could contribute to reduced adaptive B- and T-cell responses to parasite infections and vaccines [49–51]. The persistently high IL-10, and its positive association with IFN-γ, CCL17 and CCL18 indicated that IL-10 remained a major modulating component for cellular activation and immune regulation.

### O. volvulus parasite transmission by Simulium damnosum s.l.

The anti-vectorial control measure applied over decades by the OCP have reduced ABR and ATP in the Mô river basin in central Togo, and with annaul mass drug application of ivermectin the prevalence of *O*.*volvulus* mf in the village populations was reduced consistently to below 5% [22]. However, parasite transmission has never been interrupted completely in central Togo, and the Mô river basin remained a special intervention zone where aerial larvicide application and biannual MDA with ivermectin was applied until the end of 2012 [52]. From 2015 until 2020 a high ABR was observed in the Mô river basin similar to that observed before initiating ivermectin treatment (Fig 4), and previous surveys [22] and the present positive rtPCR results confirm ongoing transmission (Table 5).

The *O*. *volvulus* vectors *S*. *damnosum s*.*l*. were captured weekly at the river basin of Mô, catches were highest in July and August, and lowest in March and April, reflecting the seasonal abundance *S*. *damnosum* during rainy seasons. However, prevalence of *O*. *volvulus* specific Ov150 and Ov33 DNA in blackflies was highest in December and such intense transmission may be attributed to changed human riverine activities or an increased anthropophilic behaviour of blackflies [53,54]. The transmission of *O*. *volvulus* can be interrupted when the prevalence of *O*. *volvulus* in blackflies is less than 0.05% (upper 95% CI) [55], but with 0.58% in 2018 and 0.45% in 2019 the presence of Ov150 DNA in blackflies was above the WHO guidelines. Thus, *O*. *volvulus* mf-positive individuals remain in the study area, and this could be due to an incomplete ivermectin treatment coverage of communities or repeated temporary absence of mf-positive cases during MDA [22]. Furthermore, we have tested for Ov33 DNA, which encodes for a protein found in both *O*. *volvulus* and *O*. *ochengi*, and Ov33 was detected in *S*. *damnosum*. The Ov150 positive blackflies confirm *O*. *volvulus* transmission, while Ov33 DNA is present in *O*. *volvulus* and other *Onchocerca* species. Blackflies positive for Ov33 and negative for Ov150 DNA may transmit filariae of deer and bovids. Blackflies which were Ov33 positive, but Ov150 negative were likely infested with *O*. *ochengi*. In humans, *O*. *ochengi* will not develop but repeated exposure to the infective larvae of bovine *O*. *ochengi* will stimulate cross-reactive antibody with *O*. *volvulus* [56,57], and this can bias the immuno-surveillance of onchocerciasis.

## Conclusions

Thirty years of repeated ivermectin medication eliminated permanently microfilariae of *O*. *volvulus* and prevented the emergence of patent infection despite ongoing low-level parasite transmission in the study area. In mf-negative onchocerciasis patients, their parasite-specific cytokine and chemokine responses increased, while their specific antibody responses diminished to levels found in infection-free endemic controls, and their innate cellular responsiveness also declined. In the elderly patients the latter could contribute to reduced adaptive immune responses to parasite infections and vaccines. Increased chemokine responses in mf-negative onchocerciasis patients could reflect effector cell activation against tissue invasive larval stages of *O*. *volvulus*. The annual *Simulium damnosum* s.l. biting rate observed in the Mô river basin was similar to that observed before initiation of MDA with ivermectin, and the rtPCR results presented here confirm ongoing *O*. *volvulus* transmission.
